# Wikipedia and Medicine: Quantifying Readership, Editors, and the Significance of Natural Language

**DOI:** 10.2196/jmir.4069

**Published:** 2015-03-04

**Authors:** James M Heilman, Andrew G West

**Affiliations:** ^1^Faculty of MedicineDepartment of Emergency MedicineUniversity of British ColumbiaVancouver, BCCanada; ^2^Verisign Labs (Verisign, Inc.)Reston, VAUnited States

**Keywords:** health information systems, consumer health information, information sharing, information networks, information science, Internet, Web 2.0, cooperative behavior

## Abstract

**Background:**

Wikipedia is a collaboratively edited encyclopedia. One of the most popular websites on the Internet, it is known to be a frequently used source of health care information by both professionals and the lay public.

**Objective:**

This paper quantifies the production and consumption of Wikipedia’s medical content along 4 dimensions. First, we measured the amount of medical content in both articles and bytes and, second, the citations that supported that content. Third, we analyzed the medical readership against that of other health care websites between Wikipedia’s natural language editions and its relationship with disease prevalence. Fourth, we surveyed the quantity/characteristics of Wikipedia’s medical contributors, including year-over-year participation trends and editor demographics.

**Methods:**

Using a well-defined categorization infrastructure, we identified medically pertinent English-language Wikipedia articles and links to their foreign language equivalents. With these, Wikipedia can be queried to produce metadata and full texts for entire article histories. Wikipedia also makes available hourly reports that aggregate reader traffic at per-article granularity. An online survey was used to determine the background of contributors. Standard mining and visualization techniques (eg, aggregation queries, cumulative distribution functions, and/or correlation metrics) were applied to each of these datasets. Analysis focused on year-end 2013, but historical data permitted some longitudinal analysis.

**Results:**

Wikipedia’s medical content (at the end of 2013) was made up of more than 155,000 articles and 1 billion bytes of text across more than 255 languages. This content was supported by more than 950,000 references. Content was viewed more than 4.88 billion times in 2013. This makes it one of if not the most viewed medical resource(s) globally. The core editor community numbered less than 300 and declined over the past 5 years. The members of this community were half health care providers and 85.5% (100/117) had a university education.

**Conclusions:**

Although Wikipedia has a considerable volume of multilingual medical content that is extensively read and well-referenced, the core group of editors that contribute and maintain that content is small and shrinking in size.

## Introduction

Wikipedia is a multilingual, online, open-source encyclopedia that anyone with Internet access can edit. It is available in more than 275 languages and contains more than 32 million articles across a tremendously broad topic space [1]. Although a considerable amount is known about the volume of content, readership, and editor population of Wikipedia as a whole, less is known about these aspects as they pertain to Wikipedia articles in the medical domain. Moreover, non-English language editions are dramatically understudied in comparison to the larger and more popular English version.

In January of 2014, Wikipedia was referred to as “the single leading source of medical information for patients and health care professionals” by the Institute of Medical Science (IMS) Institute for Healthcare Informatics [2]. It is used as a source of health care information by 50% to 70% of physicians [3,4] and has been reported as being the single most used resource by medical students (94%) [5]. A 2013 US survey found people spend more than 52 hours a year searching for health information online, with 22% reporting using Wikipedia [6]. Wikipedia’s readership is also affected by current events, including popular culture [7] or disease outbreaks [8,9]. Because Wikipedia’s health content is extensively read by the general public and in communities of practice, its authorship and reliability are important qualities. Additionally, quantifying topic popularity can help focus improvements toward greater impact.

With respect to measures of quality, the small amount of available research came to differing conclusions [10]. In 2 small samples, Wikipedia’s accuracy was found to be similar to that of UpToDate, eMedicine, and the National Cancer Institute’s Physician Data Query (PDQ) comprehensive cancer database [10]. A narrow look at pharmacological articles assessed Wikipedia’s accuracy to be high based on significant overlap with textbook sources [11]. Other research found a selection of 50 English medical articles to be relatively well cited [12]. Since 2010, the number of health science academic articles using Wikipedia as a citation has increased substantially [13]. Differing research has found Wikipedia’s coverage to be incomplete or less than that of professional sources [10]. A paper examining gastroenterology articles from 2013 found insufficient discussion of the mechanisms of disease [14]. A comparison of pediatric otolaryngology articles between Wikipedia, MedlinePlus, and eMedicine found Wikipedia had a similar accuracy to MedlinePlus, but less than that of eMedicine [15].

In our subsequent analysis, we will report on the amount of medical content on Wikipedia. This includes determining the number of references supporting this content and how this quantity has changed over the past 5 years. Readership for both English and non-English versions in 2013 will be analyzed, along with an attempt to determine how the popularity of Wikipedia’s medical content compares to that of other well-known Internet health care sites. We will determine if the most commonly viewed articles are those that cover major global health problems or more obscure ones. Finally, the size and makeup of the core editor community will be examined, including how this has changed since 2009.

## Methods

### Amount of Wikipedia Medical Content

To quantify the number of medical articles and the amount of content within them, one must first determine the subset of Wikipedia which is medically relevant. Wikipedia has a category hierarchy that is built collaboratively, similar to how its core content is amassed and refined. These categories are the basis for identifying medical articles, drawn from the tagging work of WikiProject Medicine [16], which identified those English articles that fall within its project’s scope.

Examples of medical articles include medical diseases and syndromes, medical procedures and diagnostic tests, medications and drugs, and articles related to the history of medicine. Some fitness, pathogenic, and microbiology topics are also categorized as medical; notable health care workers also often meet the threshold. However, articles for anatomy, individuals with specific conditions, pharmaceutical companies, and hospitals tend not to be categorized as “medical” because they are usually well covered by other projects [17].

To identify non–English language equivalents for English articles we relied on the interlanguage link infrastructure. Also collaboratively built, these links build a graph of all articles—across all language editions—corresponding to a shared topic. Before 2013, these links were annotated in the articles themselves in a distributed fashion. Throughout 2013, these links were migrated to a centralized location (WikiData) for ease of maintenance. When we measured the amount of content (in bytes) we accounted for this migration otherwise it would appear articles were losing content when, in fact, duplicate content was just being more efficiently stored.

Determining the size of a language’s medical article membership was straightforward aggregation. Our analysis reports only on article content, not the discussion or policy-based pages that surround it. Programmatic access to category and interlanguage data are available via the Wikimedia application program interface (API) [18]. That same API permitted us to obtain an article’s full content at any historical timestamp. We used snapshots from start-2013 and end-2013 to plot the byte growth of medical content, measuring only textual content in this manner.

### Citations Supporting Wikipedia’s Medical Content

One marker to estimate the quality of Wikipedia’s content is the number of references present in articles and the reputation of those referenced sources. Leveraging the ability to obtain an article’s full content at any timestamp, we parsed that content for standardized citation templates (ie, the “<ref>” and “{{cite}}” notations). Counting template usage is straightforward and article snapshots at end-of-year 2009-2013 were used to analyze longitudinal trends. The citation templates also contained a “source” field. We used this to analyze the relative citation counts of leading medical journals, bearing in mind that nonstandardized naming and abbreviation conventions (eg, New England Journal of Medicine, NE Journal of Medicine, NE J Med) inhibit precise aggregation. In particular, we highlighted citations to Cochrane reviews because they are a highly regarded source. Parsers based on regular expressions were used in reference counting and source extraction.

### Readership of Wikipedia’s Medical Content

Readership of specific articles and medical content in total were derived from the hourly page view aggregates [19] made available by the Wikimedia Foundation (WMF). These are large plaintext files in which each line contains a language, article title, and view count—with a single day’s volume (24 files) on the order of 10 GB in size. We authored scripts to obtain and process these files nightly, writing daily aggregates to a persistent database table indexed by language and article.

These files report only “desktop” views. However, mobile views were reported at project-scale [1,20] (eg, for all of English/French/Spanish Wikipedia), permitting some rough estimates if one assumed mobile traffic was uniformly proportional across all articles. An examination of the phenomena underlying this collection and broader readership trends was done by West [7].

Our database of daily views can be queried to produce aggregates by language, specific article, or the topics that span multiple language equivalents. To compare Wikipedia’s medical readership to that of other common health care websites, we used SimilarWeb [21], a traffic measurement service. We multiplied the “estimated visitors” and “page views per visit” metrics that the service provides to produce a page views statistic comparable to the one reported by the WMF.

To measure topic readership variance between languages we identified a core set of equivalent articles that existed in all Wikipedia’s 10 largest language editions. We first analyzed these by topic, finding anomalous popularity patterns and outliers. For an aggregate comparison, we also calculated the Pearson correlation coefficient between all language pairs.

We also wanted to determine if diseases of greater global severity were more frequently viewed Wikipedia topics. To do so, we took the top 20 diseases by disability adjusted life years (DALYs) and the top 20 diseases by years lived with disability (YLDs) for 2012 as reported by the World Health Organization [22], yielding 33 conditions in combination. We then found the 42 corresponding English Wikipedia articles for each disease (some, such as “child behavioral disorders” referred to both “ADHD” and “conduct disorder”). Traffic on these articles was compared against that on a broader set of Wikipedia articles corresponding to diseases, as identified by the presence of a standardized template (“infobox”) that concisely summarized disease metadata (eg, a condition’s index in various disease databases).

### Quantity/Characteristics of Wikipedia’s Medical Contributors

Already leveraged for categories and language links, the Wikimedia API also permits one to crawl version histories to gather metadata about an article’s editors. Aggregating this across all medical articles (or just those of a particular language), we were able to plot participation at various thresholds. In particular, we identified 274 contributors who made more than 250 edits to medical articles in 2013. In May 2014, we utilized a Wikipedia messaging system to award 271 of these users a “barnstar,” a digital form of peer-to-peer recognition. Posted to users’ talk pages, the awards contained a request to complete a survey containing 6 questions:

1. What is your highest level of education?
2. Do you currently work in the health care field? Or have you previously?
3. Are you currently studying health care (a student)?
4. What language of Wikipedia do you mostly work on?
5. Did you receive a barnstar?
6. How do you identify your gender?

Question #5 was used to sanity check respondents (because barnstar awards are public, uninvited participants could traverse the survey link). We also posed an open question: “Why do you edit Wikipedia’s medical content?”

## Results

### Amount of Wikipedia Medical Content

#### Number of Articles

**Figure 1:**
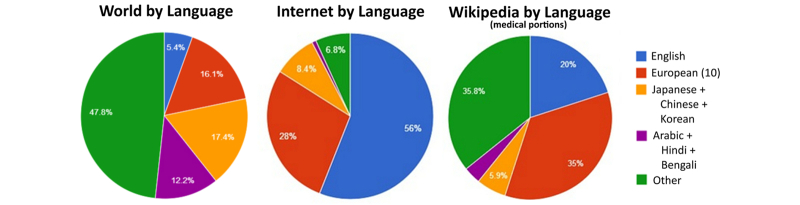
Relative amount of population/content by natural language group. The 10 European languages are German, French, Spanish, Polish, Italian, Portuguese, Russian, Dutch, Swedish, and Catalan.

Wikipedia had 155,805 medical articles across 255 natural languages at the end of 2013. A further 31 languages did not contain any medical articles per our methodology. Of the more than 155,000 articles, 29,072 (18.66%) were in English. Although a significant portion of Wikipedia’s content (both medical and otherwise) is in English, this imbalance is less than that observed across the broader Internet (Figure 1). In Figure 1, the “world by language” subgraph was based on 2007-2010 data per the aggregation of the Wikipedia community [23], “Internet by language” was derived per W^3^Techs Web Technology Surveys [24], and “Wikipedia by language (medical portions)” was based on independent calculations of medical articles by language edition. Note that for all independent calculations/figures/graphs presented in this paper, Multimedia Appendix 1 presents raw data and/or extends those presentations.

**Figure 2:**
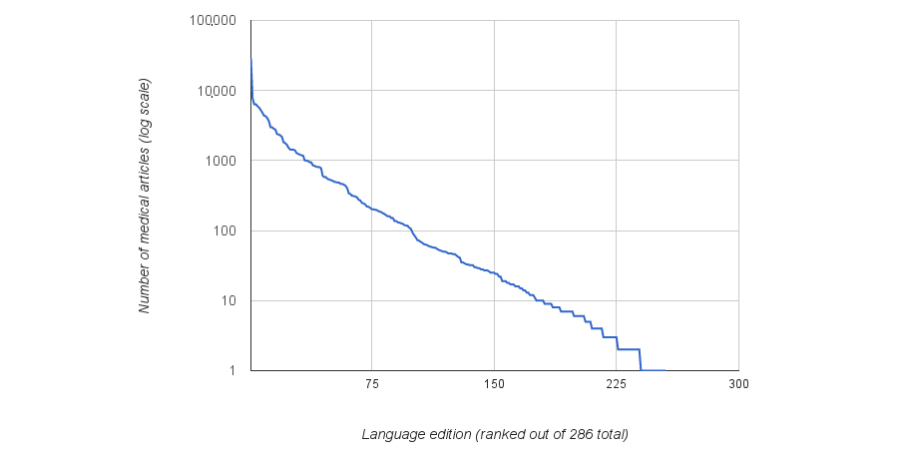
Distribution for the quantity of medical articles in a Wikipedia language edition presented in rank order (note log scale on y-axis).

Table 1 presents the top languages by quantity of medical articles. Going beyond this list, the top 10 languages made up 51.37% (80,043/155,805) of the total articles, whereas the top 25 languages accounted for 74.97% (116,808/155,805). Figure 2 plots the article quantity distribution, showing it to have a power-law distribution (ie, few languages have many articles and vice versa).

**Table 1:** Wikipedia language editions ranked by number of medical articles and the amount of textual content in each language (in bytes).

Rank	Language	Medical articles, n	Size (MB)
1	English	29,072	241
2	German	7761	66
3	French	6372	57
4	Spanish	6367	51
5	Polish	5999	28
6	Italian	5677	44
7	Portuguese	5269	25
8	Russian	4832	48
9	Dutch	4391	18
10	Japanese	4303	36
11	Arabic	4055	26
12	Swedish	3661	12

#### Bytes of Content

**Figure 3:**
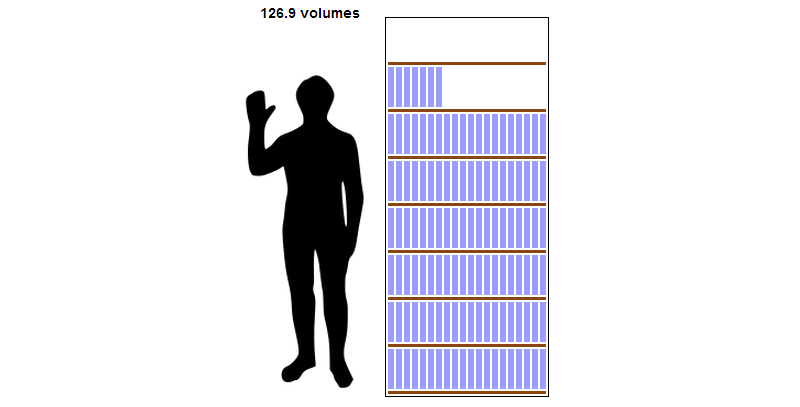
Estimated volume of Wikipedia’s medical content if printed (attribution of human outline: Linda Salzman Sagan).

At the end of 2013, Wikipedia had 1016 MB of textual medical content, up 10.19% from 1 year earlier when the total was 922 MB. English medical articles saw the most growth during this period, gaining some 19.7 MB. Assuming the average word has 6 characters, this equates to 3.28 million English words added in 2013. If the total (combined language) 1016 MB of content were printed in textbooks roughly the size of the *Encyclopedia Britannica* at 8 million characters per volume, it would consume 126.9 volumes (Figure 3). English-language medical articles were responsible for 23.72% (241/1016 MB) of all medical content (by bytes). The next largest languages per this metric were German, French, Spanish, Russian, Italian, Japanese, Polish, Arabic, and Portuguese (similar but not identical to Table 1). Together the top 10 languages accounted for 61.22% (622/1016 MB) of all byte content.

### Citations Supporting Wikipedia’s Medical Content

**Figure 4:**
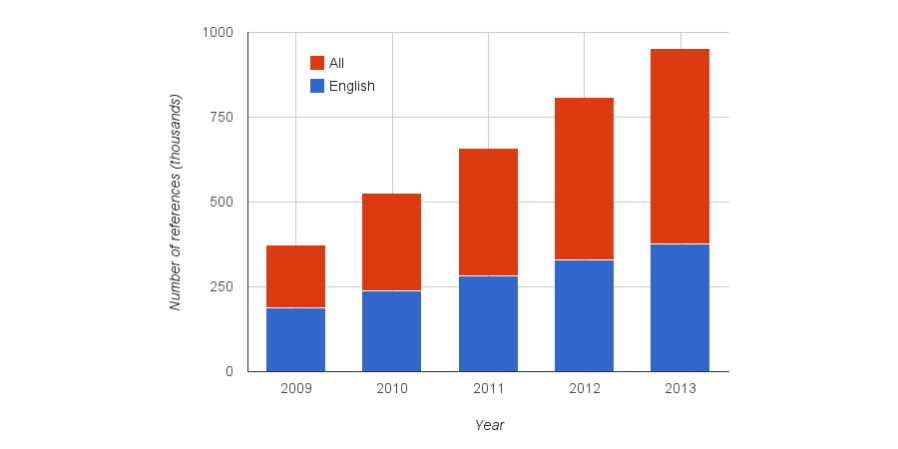
Citations/references appearing in the medical content of English Wikipedia and all Wikipedia languages based on year-end snapshots.

As a marker for Wikipedia’s reliability, we counted the number of references in year-end article versions between 2009 and 2013. This was done for medical portions of both English Wikipedia and all languages (Figure 4). We found that English references more than doubled from 187,107 to 376,123, whereas the increase was more than 2.5 times from 373,558 to 952,053 across all languages. Note that this citation growth ratio significantly outpaced that observed for byte growth.

**Figure 5:**
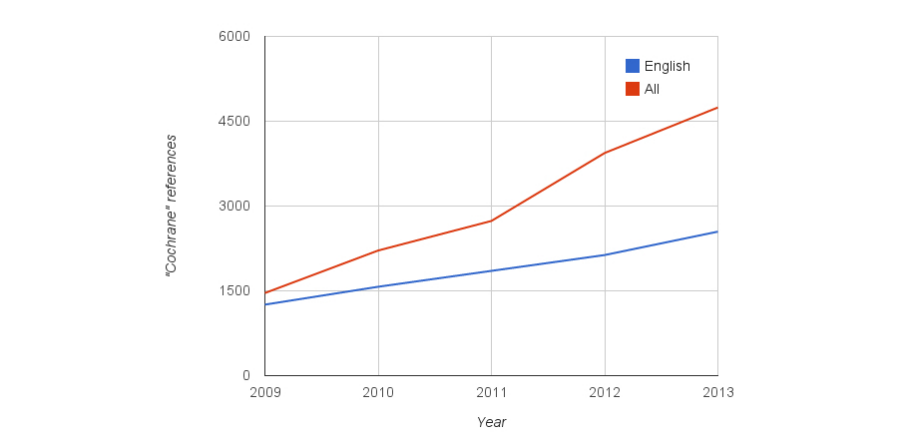
References to “Cochrane (reviews)” in medical content of English Wikipedia and all Wikipedia languages in both plain text and citation formats.

By parsing a standardized citation format, we were able to determine the journals that were most commonly used as references on Wikipedia were also some of the most respected, including *The Lancet*, *The New England Journal of Medicine*, *Nature*, *British Medical Journal*, *JAMA*, *Science*, and the Cochrane Database of Systematic Reviews. Although a lack of standardized naming/abbreviation conventions prevented precise aggregation, we were able to measure references to a high-quality source. Plain text and citation references to “Cochrane (reviews)” (Figure 5) across all languages increased nearly 3-fold from 2717 in 2009 to 7290 in 2013.

### Readership of Wikipedia’s Medical Content

#### Comparison Between Wikipedia and Other Health Care Websites

Before embarking on traffic comparisons between Wikipedia and other health care sites, we first established Wikipedia’s medical readership in isolation. In 2013, across all languages, Wikipedia’s medical content received 4.88 billion nonmobile views (estimates put the mobile-inclusive total close to 6.5 billion). Approximately 4.56 billion of these were in the top 12 languages (Table 2), with English accounting for 46.72% (2.28/4.88 billion views).

**Table 2:** Languages sorted by millions of page views to medical content in 2013 and percentage of medical views out of all language views.

Language	Medical page views (million), n	All language views (million), n (%)
English	2277	91,252 (2.49)
Spanish	659	14,806 (4.45)
German	348	11,067 (3.15)
Japanese	254	12,535 (2.02)
French	219	8305 (2.63)
Portuguese	212	5266 (4.03)
Russian	150	12,072 (1.24)
Polish	147	5691 (2.59)
Italian	147	5738 (2.56)
Dutch	76	2198 (3.44)
Chinese	36	3775 (0.96)
Turkish	34	1646 (2.06)

Medical content accounted for 0.64% (0.029/4.5 million) of all articles on English Wikipedia, yet these received 2.49% (2277/91,252 million) of all English Wikipedia page views. Similar patterns were observed across many language editions, with medical articles receiving far more than the mean expected traffic. As a portion of all content, among prominent languages, medical readership varied from 0.96% (36/3775 million) in Chinese to 4.45% (658/14,806 million) in Spanish; the global percentage across all languages was 2.50% (4.88/195 billion), roughly the same as for English.

Recall that we used the Web monitoring service SimilarWeb [21] to estimate the traffic received at other health care websites. Despite having precise page view data for Wikipedia’s medical portions, in the interest of fairness, we also derived Wikipedia’s totals from SimilarWeb. That service’s sampling methodology likely introduces bias we would prefer to be uniform across all sites under evaluation. The health care sites we examined (National Institutes of Health, WebMD, Mayo Clinic, National Health Service, World Health Organization, UpToDate) host exclusively medical content. In contrast, the traffic statistics SimilarWeb reports for the Wikipedia domain must be scaled down to its medical portion (2.49%).

**Figure 6:**
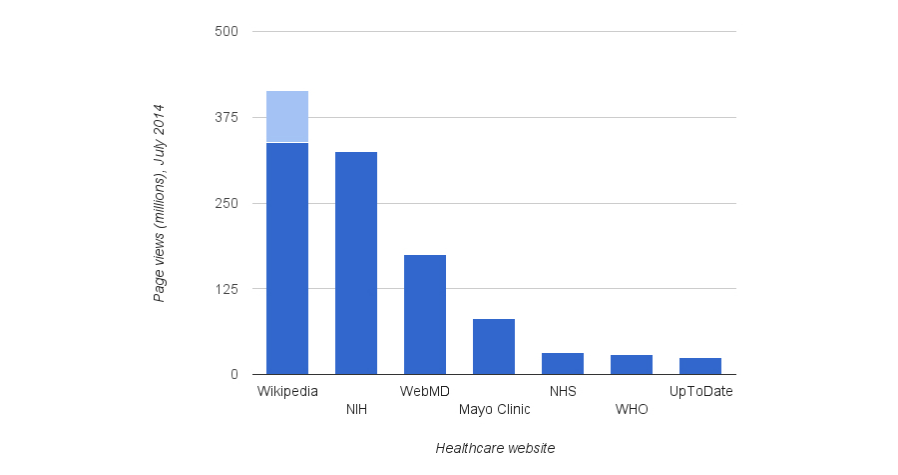
Health care site traffic comparison. Light blue portions represent official Wikimedia Foundation data.

Figure 6 presents the comparison after such adjustments for July 2014, with the light blue portion capturing that SimilarWeb slightly underreports traffic compared to the WMF data (recalling that neither reports mobile views). Regardless, Wikipedia appears to be the most utilized online health care information resource.

### Comparison Among Wikipedia’s Natural Language Editions

The popularity of individual topics across languages varied dramatically. Among the 100 most popular English articles, none were unanimously in the top 100 across 9 other prominent languages (compromising the 10 most popular languages by overall page views in 2013) in which a corresponding article existed. For example, “Down syndrome” was third most popular in German, seventh most accessed in Italian/Polish, and 17th in English. However, it was outside the top 1000 in Russian, Japanese, French, Portuguese, and Chinese. “Asperger syndrome” was 1 of few articles close to being in the top 100 most viewed in all languages, but was nearly 1500th in Russian. Similarly, “tuberculosis” fared well in all languages except French and Polish. “Sexual intercourse”—a typical stronghold of Internet attention—was only in the top 10 most popular articles for English, where it secured third place. Table 3 presents the most popular topics overall and Table 4 further highlights popularity variance.

**Table 3:** Medical topics with the most traffic summed across languages. View count is for 2013 and the number of languages with a corresponding article is presented.

Topic/Article	Views (million), n	Languages, n
Leonardo da Vinci	17.36	180
Asperger syndrome	15.48	56
Schizophrenia	13.84	74
Bipolar disorder	13.12	55
Sexual intercourse	13.07	96
Tuberculosis	11.86	123
Diabetes mellitus	11.28	113
Autism	10.43	80
HIV/AIDS	9.65	128
Angelina Jolie^a^	9.51	98
Human	9.47	165
Hemorrhoid	9.42	73
Pneumonia	9.26	90
Blood type	9.20	67
Human papillomavirus	8.97	39
Down syndrome	8.83	66

^a^ The full article title was “Angelina Jolie Cancer Treatment,” which was a newsworthy topic of 2013. However, that title redirected to a subsection of the “Angelina Jolie” article, effectively making her (broadly popular) article and its (broadly popular) foreign language equivalents part of the medical category, despite the fact many visitors likely arrived there for nonmedical reasons.

**Table 4:** Topics having most and least variable popularity rank across the top 10 languages.

Topic/Article	Relative variance^a^	Most popular (rank)^b^	Least popular (rank)^b^
Down syndrome	1.000	German (3)	Russian (1506)
Pneumonia	0.973	Russian (1)	German (1516)
Diabetes mellitus	0.964	Spanish (6)	French (1522)
Hypertension	0.953	Chinese (4)	Italian (1522)
Myocardial infarction	0.937	Russian (17)	Polish (1484)
Sixth disease	0.022	Spanish (1012)	English (1402)
Chitosan	0.021	Chinese (609)	Spanish (903)
Candidiasis	0.019	Portuguese (81)	Polish (394)
Mortality rate	0.019	Italian (408)	Japanese (729)
Asbestos	0.005	German (35)	Portuguese (187)

^a^ Relative variance is the percentage of the maximum observed variance (ie, it is not an absolute measure, but based on the variance calculated for the “Down syndrome” article).

^b^ Popularity rank goes from 1 (most popular) to 1536 (least) because there were 1536 articles in all of the 10 languages used herein; a constraint that helped to normalize these comparisons.

Although sometimes regional or cultural trends were observed (eg, disease effected regions having high popularity for the corresponding article in the local language), a broader explanation of these patterns is a topic for future investigation.

Rather than looking at articles or topics in isolation, we calculated rank similarity between language pairs (Table 5). Working from the set of topics with articles in all the top 10 language editions, we found Portuguese and Spanish visitors (*r*=.668) had the most similar browsing habits, whereas Russian and English visitors (*r*=.207) were most dissimilar.

**Table 5:** Pearson correlation coefficient (eg, “rank similarity”) metric for medical topic popularity in 10 prominent languages.^a^

Language	English	Spanish	Russian	Japanese	German	French	Portuguese	Italian	Polish	Chinese
English	1	.313	.207	.263	.334	.294	.305	.287	.315	.286
Spanish		1	.423	.412	.555	.584	.668	.598	.564	.471
Russian			1	.332	.425	.408	.410	.426	.439	.372
Japanese				1	.529	.470	.412	.479	.486	.532
German					1	.613	.551	.639	.642	.504
French						1	.563	.633	.588	.464
Portuguese							1	.585	.556	.439
Italian								1	.608	.484
Polish									1	.488
Chinese										1

^a^ More informally: “how similar is the popularity ordering for topics between 2 languages?” The measure is symmetric.

### Correlation of Wikipedia Article Traffic and Disease Prevalence

A 2014 IMS report made the claim that “rarer diseases, which often have fewer available information sources and are less understood by patients and clinicians, show a higher frequency of [Wikipedia] visits than many more common diseases” [2]. Given that English is frequently the language used to search for information on Wikipedia regardless of a person’s country of origin, we used the English traffic data to gain perspective on this claim. We found that the articles associated with the 20 conditions having the greatest YLD and the 20 conditions with the greatest DALYs had an average view count of 1.68 million in 2013. This compares to an average of 189,351 views for the 4791 articles tagged with the disease “infobox” and 78,000 views for the average English medical article. Clearly, globally prevalent and well-known medical conditions tend to receive considerable traffic.

Such macroscale correlation is intuitive, but recent research [9] has also demonstrated the more nuanced capability to utilize traffic data for individual articles in near real time. That work found that the popularity of influenza articles not just correlated with the spread of the disease, but could also be temporally analyzed to create reasonably accurate infection forecasts. The extent to which this applies across the entire article base and the ways the health care community can utilize such rapid signaling are topics for future work.

### Quantity/Characteristics of Wikipedia’s Medical Contributors

#### Year-Over-Year Analysis of Editor Numbers

Given Wikipedia’s collaborative nature, it is logical to investigate the editor community that has authored the content of such a frequently accessed resource. Most often, “editors” in this context are users with a persistent account name and log-in credentials. Although one may edit without an account, rarely do such users exhibit the consistent participation on which we focused. Of the 274 top contributors, just 4 edited without an account name.

We measured participation by looking at an editor’s quantity of contributions on medical articles in a given calendar year. The following are some participation thresholds measured across all languages in 2013: ≥5 edits=21,563 editors; ≥25 edits=5573 editors; ≥100+ edits=1237 editors; ≥250 edits=274 editors; ≥1000 edits=39 editors; ≥10,000 edits=1 editor (this paper’s lead author). There were 32 language versions that had at least 1 editor with ≥250 edits in 2013.

**Figure 7:**
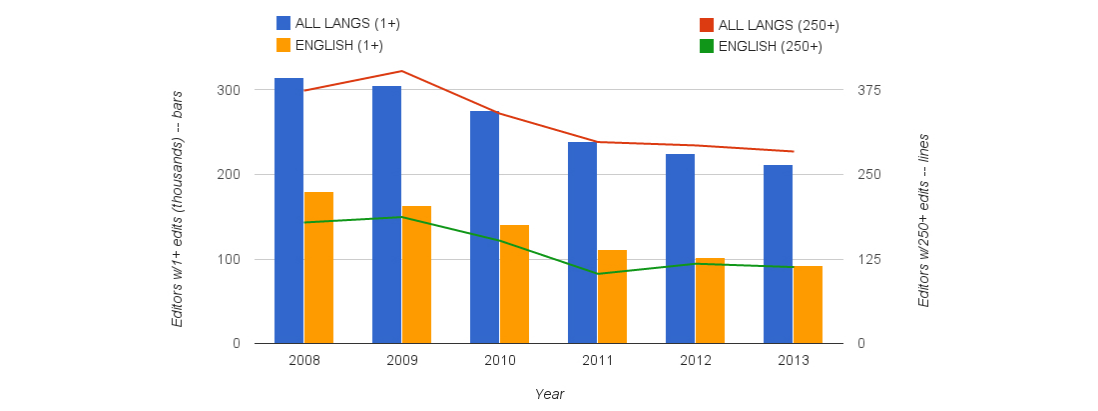
Quantity of editors making ≥1 and ≥250 medical contributions by year across all languages for English and all languages.

We plotted some of these same thresholds on a yearly basis from 2008 to 2013 with breakdowns (Figure 7). We found that at all participation thresholds the number of editors decreased. Over this 5-year span, the decrease in editor numbers was approximately 40% for English Wikipedia, with 10%-20% attrition typical for non-English languages.

Not included in the preceding totals is the work of nonhuman, automated “bot” editors: computer programs that perform much repetitive maintenance. Bots and humans combined made 1,106,575 medical edits in 2013 with 406,003 (36.69%) of those in English. Bots accounted for 24.72% (274k/1107k) of the global total and 10.54% (43k/406k) of the English total, numbers slightly inflated due to the bot-driven migration of interlanguage links as described in the Methods section.

#### Contributor Demographics/Background via Survey

In May of 2014, we sent out a survey to 271 of the 274 top medical editors in 2013. Three users were omitted because they had been blocked from contributing to Wikipedia due to various issues. Of these, 117 (43.2%) responded and their answers are summarized in Table 6.

We found more than half of editors (50.4%, 59/117) were either health care professionals or studying health care. Of the 58 outside of health care, 17 used the open text area to describe their activities as primarily grammatical, formatting, language simplifications, and the removal of vandalism. Fifteen others reported more substantive editing despite lacking formal medical training. In some cases (2 self-reported), contributors were arguably experts despite not being health care providers: 1 was a PhD biochemist and another was a SCUBA diver editing in related medical spaces.

**Table 6:** Survey responses from 117 top medical editors across all language editions.

Question		n (%)
**What is your highest level of education?**		
	High school or less	17 (14.5)
	Bachelor’s	39 (33.3)
	MS/PhD/MD	61 (52.2)
**Do you currently work in the health care field? Or have you previously?**		
	Yes	59 (50.4)
	No	58 (49.6)
**Are you currently studying health care (a student)?**		
	Yes	17 (14.5)
	No	100 (85.5)
**What language of Wikipedia do you mostly work on?**		
	English	58 (49.6)
	Non-English	59 (50.4)
Did you receive a barnstar? (yes)^a^		117 (100)
**How do you identify your gender?**		
	Male	96 (82.1)
	Female	10 (8.5)
	Other/omitted	11 (9.4)

^a^ Sanity check; surveys with “no” responses were discarded.

## Discussion

### Principal Results

Wikipedia’s medical content is made up of more than 155,000 articles and 1 billion bytes of text across 255 languages. This content is supported by more than 950,000 references and was viewed more than 4.88 billion times in 2013 (with mobile-inclusive estimates at 6.5 billion). Third-party analytics suggests Wikipedia is the most viewed medical resource globally. As of 2013, the core editor community numbered less than 300 and had decreased over the previous 5 years. The members of this community are half health care providers and 85% have a university education.

### Limitations

#### Amount of Wikipedia Medical Content

Our analysis depended heavily on the Wikipedia editor community to establish (1) what constitutes a medically related article and (2) the interlanguage links between corresponding articles. Whether or not something is related to medicine or related “enough” to justify a tagging is a subjective distinction. Interlanguage links are often less ambiguous, but still require a bilingual speaker who is familiar with Wikipedia syntax.

Although subjectivity might shift these bounds slightly, more articles have likely never been considered in these contexts, either because they are undiscovered entirely or they are too emergent, tangential, or unpopular to draw the attention of the editors who typically make category and interlanguage annotations. Although usually quickly restored [25], “vandals” also sometimes destroy tags or links with malicious intent.

Following the very nature of collaborative work, it is our subjective experience that “major” topics are more likely to be correctly tagged and linked than more obscure ones. Thus, tagging and linking inaccuracies likely have a greater impact on article quantity measurements than readership totals. In particular, categorization omissions could be estimated by searching English Wikipedia using a database of terms such as the International Classification of Disease (ICD-10) and verifying that corresponding articles have been appropriately tagged. We leave this as a topic for future research.

Lastly, our analysis used tagged English articles as the starting point for interlanguage link discovery. A medical topic that did not have a corresponding English article version would not be included in our analysis.

#### Citations Supporting Wikipedia’s Medical Content

Wikipedia strives for verifiable content rather than the less agreeable notion of absolute “truth.” As such, information drawn from reputable sources upholds the notability and verifiability requirements that Wikipedia promotes.

In this work, we quantify the number of references (and highlight some particularly well-reputed sources) as a proxy for reliability. We recognize that the number of references is just 1 mark of quality. Content may be inaccurate despite having a citation and vice versa. Our data do not look at whether or not the text of Wikipedia accurately reflects the sources in question or if the sources are outdated. Both would be interesting questions to investigate further.

#### Readership of Wikipedia’s Medical Content

Language-scale aggregates regarding Wikipedia readership are influenced by the number of member articles. Thus, previously discussed limitations surrounding category tagging and interlanguage links also cascade into this analysis.

It is important to emphasize that none of our traffic data (Wikipedia or third party) includes readership from mobile devices. These shortcomings in the WMF’s collection infrastructure were remedied during our writing in October 2014; mobile readership will be analyzed in future work. Although allowing for fair comparison, this also means we underreport the scale at which other online health care resource operate. Across all English Wikipedia (not just medical portions), mobile views are more than 30% of the total traffic and growing [20]. Thus, readership as we present it may underrepresent the browsing habits of certain economies, languages, and regions (eg, where mobile networks are the only means of connectivity and/or cellular devices are the only affordable means of access) or certain demographics (eg, youth demonstrating a preference for mobile browsing).

Moreover, when comparing Wikipedia’s medical readership to other health care websites, one must be mindful of the varying coverage and scope. Although it would be interesting to compare per-topic page views, alternative sites (some proprietary) have not made such granular traffic data publicly available.

In our broad comparison of readership on health care websites, we relied on the third-party service SimilarWeb [21,26-32]. That service’s measurement methodology and accuracy is not known. However, it is reassuring that SimilarWeb’s page view estimates for the entirety of English Wikipedia differed only by about 3% from the more authoritative data published by the WMF.

To some extent, all information sources find themselves mirrored across the Internet and combined into other sources. However, this occurs more frequently with Wikipedia and government sources because they are freely licensed or in the public domain which encourages reuse. Such transitive/downstream consumption (both online and offline) is difficult to quantify. For example, low-cost “alternative textbook” provider Boundless amasses such open-source content when compiling its texts [33], with some becoming popular in practice [34]. Further, the National Institutes of Health (NIH) and Wikipedia often see their content integrated directly into Google search results and these sources often have high search-engine ranking [35].

#### Quantity/Characteristics of Wikipedia’s Medical Contributors

Our survey to medical editors had a response rate of approximately 43%. This raises the concern that those with the time and willingness to complete the questionnaire are somehow nonrepresentative. Although approximately half of recipients primarily edit a non-English Wikipedia, our survey was available only in English, potentially limiting and biasing the response pool. Our validation question (“Did you receive a barnstar?”) also takes respondents at their word in addition to trusting the feedback received for all other questions.

We identified 4 Internet Protocol (IP) accounts that made more than 250 edits assuming that those IP addresses are statically assigned to a single contributor. Dynamic IP assignment (ie, the Dynamic Host Configuration Protocol, DHCP) is common in residential and wireless networks and could have effects such that multiple human users inhabit a single IP over time (causing an overestimation on our part) or that a single user’s contributors are unknowingly spread across IP space (an underestimation).

### Comparison With Prior Work

The Introduction enumerates some of the prior research that qualitatively relates to this work. A purely quantitative point of reference comes from the parallel work of Farič and Potts [36], who also surveyed English Wikipedia’s most active medical editors. That research found 50% of those surveyed had a medical background, 70% were older than 30 years, most were male, and 75% had a college degree. All data points were quite similar to our findings, which additionally considered non-English editors.

### Conclusions

#### Amount of Wikipedia Medical Content

Although Wikipedia has a tremendous amount of medical content, it is primarily concentrated in English and a few major European languages. As a user-generated website, this reflects the populations that are willing and able to contribute. Wikipedia’s distribution of content by language, however, better matches global language popularity than the Internet does as a whole. Additionally there are ongoing efforts to improve Wikipedia’s medical coverage in non-English languages via a partnership with the not-for-profit Translators Without Borders.

#### Citations Supporting Wikipedia’s Medical Content

Wikipedia is relatively well referenced and by this marker is becoming increasingly reliable over time. Encouragingly, references to high-quality sources, such as The Cochrane Collaboration, are rising at a greater rate than references on the whole.

#### Readership of Wikipedia’s Medical Content

A previous IMS report [2] claimed that Wikipedia is the single most used medical resource on the Internet. Our statistical work herein appears to confirm this assertion, with conservative analysis putting Wikipedia’s readership on par with NIH and surpassing that of WebMD (2 sites traditionally atop the health category). With the Internet likely to be the most consulted information medium, Wikipedia may well be the most used medical resource overall.

Our study unexpectedly found strong variance (up to a factor of 4) in the proportional popularity of health content across different languages. The catalyst for this variation is unclear. Is it the case that Spanish speakers care more about their health than Chinese speakers? Or do Chinese populations prefer a different information resource?

We also found that popular topics/articles differed wildly among languages. This has interesting ramifications as emergent language editions try to expand their medical content (either organically or through translation). Simply assuming content that is well read in 1 language will draw audiences in another is insufficient and more careful cultural consideration may be prudent.

#### Quantity/Characteristics of Wikipedia’s Medical Contributors

Although Wikipedia’s medical content has tremendous readership, the number of significantly active contributors is few. It is concerning that these editor numbers, at all thresholds, have decreased over the past 5 years. This trend is one exhibited not just by medical contributors, but the overall Wikipedia community. A number of explanations have been proposed for this poor retention and recruitment: (1) deterrents such as stricter reference requirements and more policy, (2) growing competition for participant attention in the open-source and user-generated content communities, (3) xenophobia and a community unwelcoming of new users [37], and (4) the perception that in some languages there remains little “low hanging fruit” to be authored. Understanding and reversing this trend is an area of active research for Wikipedia and its subcommunities.

The community of medical editors, like Wikipedia overall, is male dominated [38]. The reasons are not entirely clear, but some possibilities include technical barriers, lack of self-confidence, minimal social activity, and the adversarial nature of some discussions [39]. Efforts to make Wikipedia more female friendly are also ongoing.

Our survey of Wikipedia’s medical contributors found many are health care professionals and most are university educated. Although just 29% of the US population has a Bachelor’s degree [40], 85% of Wikipedia’s core medical editors have attained one (with more than 50% going beyond that level). Educational levels attained were similar between editors for English and non-English versions. These educational and professional benchmarks put into doubt the claims by some that Wikipedia is “antiexpert” [41].

## Multimedia Appendix 1

Additionally data.

## Abbreviations

API: application program interface
DALY: disability adjusted life years
DHCP: Dynamic Host Configuration Protocol
IMS: Institute of Medical Science
IP: Internet Protocol
NIH: National Institutes of Health
PDQ: Physician Data Query
WMF: Wikimedia Foundation
YLD: years lived with disability

## References

[1] Zachte E (2014). Wikimedia report card-August 2014.

[2] (2014). IMS Health.

[3] Heilman JM, Kemmann E, Bonert M, Chatterjee A, Ragar B, Beards GM, Iberri DJ, Harvey M, Thomas B, Stomp W, Martone MF, Lodge DJ, Vondracek A, de Wolff JF, Liber C, Grover SC, Vickers TJ, Meskó B, Laurent MR (2011). Wikipedia: a key tool for global public health promotion. J Med Internet Res.

[4] Hughes B, Joshi I, Lemonde H, Wareham J (2009). Junior physician's use of Web 2.0 for information seeking and medical education: a qualitative study. Int J Med Inform.

[5] Allahwala UK, Nadkarni A, Sebaratnam DF (2013). Wikipedia use amongst medical students - new insights into the digital revolution. Med Teach.

[6] (2014). Makovsky Integrated Communications.

[7] West AG, Milowent (2013). Wikipedia Signpost.

[8] McIver DJ, Brownstein JS (2014). Wikipedia usage estimates prevalence of influenza-like illness in the United States in near real-time. PLoS Comput Biol.

[9] Generous N, Fairchild G, Deshpande A, Del Valle SY, Priedhorsky R (2014). Global disease monitoring and forecasting with Wikipedia. PLoS Comput Biol.

[10] Mesgari M, Okoli C, Mehdi M, Lanamäki A (2014). “The sum of all human knowledge”: A systematic review of scholarly research on the content of Wikipedia. Journal of the Association for Information Science and Technology.

[11] Kräenbring J, Monzon PT, Gutmann J, Muehlich S, Zolk O, Wojnowski L, Maas R, Engelhardt S, Sarikas A (2014). Accuracy and completeness of drug information in Wikipedia: a comparison with standard textbooks of pharmacology. PLoS One.

[12] Haigh CA (2011). Wikipedia as an evidence source for nursing and healthcare students. Nurse Educ Today.

[13] Bould MD, Hladkowicz ES, Pigford AE, Ufholz L-A, Postonogova T, Shin E, Boet S (2014). References that anyone can edit: review of Wikipedia citations in peer reviewed health science literature. BMJ.

[14] Azer SA (2014). Evaluation of gastroenterology and hepatology articles on Wikipedia: are they suitable as learning resources for medical students?. Eur J Gastroenterol Hepatol.

[15] Volsky PG, Baldassari CM, Mushti S, Derkay CS (2012). Quality of Internet information in pediatric otolaryngology: a comparison of three most referenced websites. Int J Pediatr Otorhinolaryngol.

[16] Wikipedia.

[17] (2014). Wikipedia.

[18] Wikipedia.

[19] Page view statistics for Wikimedia projects.

[20] Zachte E (2014). Page views for Wikimedia, all projects, all platforms, normalized.

[21] (2014). SimilarWeb.

[22] World Health Organization.

[23] (2014). Wikipedia.

[24] (2014). W3Techs Web Technology Surveys.

[25] West AG (2013). Damage Detection and Mitigation in Open Collaboration Applications [PhD thesis].

[26] http://www.similarweb.com/website/en.wikipedia.org.

[27] http://www.similarweb.com/website/nih.gov.

[28] http://www.similarweb.com/website/webmd.com.

[29] http://www.similarweb.com/website/mayoclinic.org.

[30] http://www.similarweb.com/website/nhs.uk.

[31] http://www.similarweb.com/website/who.int.

[32] http://www.similarweb.com/website/uptodate.com.

[33] Owen J (2013). Boundless.

[34] Weddell C (2014). Boundless.

[35] Laurent MR, Vickers TJ (2009). Seeking health information online: does Wikipedia matter?. J Am Med Inform Assoc.

[36] Farič N, Potts HWW (2014). Motivations for contributing to health-related articles on Wikipedia: an interview study. J Med Internet Res.

[37] Halfaker A, Kittur AN, Riedl J (2011). Don't bite the newbies: How reverts affect the quantity and quality of Wikipedia work.

[38] Hill BM, Shaw A (2013). The Wikipedia gender gap revisited: characterizing survey response bias with propensity score estimation. PLoS One.

[39] Gardner S (2011). Sue Gardner's Blog.

[40] United States Census Bureau.

[41] Goodwin J (2009). The authority of Wikipedia. http://goodwin.public.iastate.edu/pubs/goodwinwikipedia.pdf.

