# Supplementary material for: Wikipedia and Medicine: Quantifying Readership, Editors, and the Significance of Natural Language
Source: J Med Internet Res. 2015 Mar 4;17(3):e62. doi: 10.2196/jmir.4069 (PMC4376174; doi:10.2196/jmir.4069)
Supplement: Supplementary file 1 [file jmir_v17i3e62_app1.pdf]

Wikipedia and Medicine: Quantifying Readership, Editors, and the Significance of Natural Language (Appendix)

James M. Heilman -- Andrew G. West

Appendix

This appendix is intended to complement the above named paper. It does so primarily by: (1) extending the tables presented in that document, (2) providing raw data for figures presented in that document, and (3) providing additional measurements that were not presented due to space constraints.

Given the scale of these measurements, and the goal of data re-use, we choose to provide these datasets in plaintext format. Here, we provide a brief description of the datasets and link to those files. Unless otherwise indicated, datasets capture calendar year 2013 data regarding Wikipedia's medical portions:

- **Traffic to individual articles (year sums)**: A large CSV file that lists all medical articles in English. Tab intended beneath each English entry are its foreign language equivalents. For all article rows, three columns of data are provided: (1) total 2013 page views, (2) that article's views as a percentage of the medical view total in the specified language, and (3) that's article's views as a percentage of the project-scale views for the specified language. http://www.andrew-g-west.com/data/human_readable_yearly.csv.zip

- **Traffic to individual articles (monthly breakout)**: Identical in format to the above, but after the column showing total page views in 2013 there are now 12 additional columns that provide monthly

breakdowns. http://www.andrew-g-west.com/data/human_readable_monthly.csv.zip

- **Number of medical articles and page views by language:** Also calculates the average number of views per article. http://pastebin.com/D0Z8Xjwg

- **Top *n* most popular articles, by language:**
  - Top 50 for "popular languages": http://pastebin.com/uQgCHAs0
  - Top 25 for all languages: http://pastebin.com/EGTn4Ey9

- **Most popular topics (across languages):** List containing the 2000 most popular topics (page views summed across corresponding articles, for all languages where an article exists). There are three columns: (1) the corresponding English article, (2) the sum of all views, and (3) the number of languages that had a corresponding article. http://pastebin.com/7pPAQ29q

- **Top traffic variance (temporally) for English medical articles:** The 500 English medical articles whose daily page views demonstrated the most variance in 2013. Individual daily totals were provided to the variance calculation. Articles with high variance are often linked to breaking news events, Google Doodles, Reddit threads, *etc*. Moderate variance is often observed with seasonal diseases and conditions. http://pastebin.com/erLELAqf

- **Top traffic variance, measured across languages:** The 500 topics that have the most traffic variance across the 10 largest Wikipedia editions. The 10 inputs to the variance function are not view counts, but normalized view rankings (i.e., the most popular article in a language receives rank "1"). This measurement captures articles that are very popular in one/some languages, and dramatically less so in others. http://pastebin.com/SJm1p9cg. As a complement to this, we also provide the 500 topics with the least variance. http://pastebin.com/H3eAHfd5

- **Medical content size and growth, 2012-2013:** Per language breakdown of textual byte size of medical articles at year end 2012 and 2013. The impact of Wikidata link migration is also included, so that year-over-year growth can be accurately calculated while excluding that influence. http://pastebin.com/8ia4U6ab

- **Quantity of edits by language/editor thresholds:** The number of edits per language in calendar year 2013. Additional columns enumerate the number of users in that language having {1+, 5+, 10+, 25+, 50+, 100+, 250+, 500+, and 1000+} edits. http://pastebin.com/3j4CWbpS

- **Editors having 250+ med. edits in 2013:** This includes bots, whose names by rule include the "bot" substring http://pastebin.com/GJ3GU75T

- **"Cochrane" references by language/year:** http://pastebin.com/3AJK1L0X

- **Most commonly cited journals:** http://pastebin.com/euvaTiNT

- **Yearly citation growth:** http://pastebin.com/H1tA1mxX

- **Miscellaneous queries:** Some quick queries and sums that include results such as: (1) topics having a corresponding article in most language editions, and (2) project-scale view counts for 2013 (not just medical portions). http://pastebin.com/WsSnC4E2
